# Gastric Cancer in the Young: Is It a Different Clinical Entity? A Retrospective Cohort Study

**DOI:** 10.1155/2014/125038

**Published:** 2014-02-13

**Authors:** Adolfo Pisanu, Mauro Podda, Alessandro Cois, Alessandro Uccheddu

**Affiliations:** ^1^Department of Surgery, Clinica Chirurgica, Azienda Ospedaliero-Universitaria, Presidio Policlinico di Monserrato, Blocco G, SS 554, km 4,500, 09042 Monserrato, Italy; ^2^Clinica Chirurgica, University of Cagliari, Azienda Ospedaliero-Universitaria, Presidio Policlinico di Monserrato, Blocco G, SS 554 km 4, 500-09042 Monserrato, Italy

## Abstract

*Background.* The rate of gastric cancer in young patients has increased over the past few decades. The aim of this study was to search for independent risk factors related to patients of younger age. *Methods.* From January 1996 to December 2012, a series of 179 consecutive patients were admitted to our surgical department because of a gastric cancer. We carried out a retrospective cohort study in 20 patients younger than 50 and in 112 patients aged 50 and older treated by curative gastrectomy. The comparison involved the evaluation of patient and tumor characteristics. *Results. *Younger patients had significantly less comorbidities and a more favorable American Society of Anesthesiology score; they had significantly less preoperative weight loss and a significantly longer duration of symptoms; *Helicobacter pylori* infection and diffuse histological type were significantly associated with younger age. There was no statistically significant difference regarding overall and cancer-related 5-year survival; advanced cancer stage and diffuse histological type were the independent negative prognostic factors influencing cancer-related survival. *Conclusions.* We do not have sufficient evidence to consider gastric cancer in younger patients as a different clinical entity. Further studies are needed to understand carcinogenesis in younger patients and to improve gastric cancer classification.

## 1. Introduction 

Gastric cancer is deemed to be the second most prevalent cause of cancer-related death, with the highest incdence in middle-aged and elderly populations [1, 2]. The rate of gastric cancer in young patients has increased over the past few decades, despite a reduction in the overall prevalence of the disease [3, 4]. Although gastric cancer is rare in young persons, it is commonly believed that it exhibits more aggressive biological behavior in these patients and worse prognosis results [5–7]. In contrast, other authors have reported that young gastric cancer patients have comparable tumor staging and survival to older patients [8, 9].

Some characteristics of gastric cancer in young patients, such as more frequent infection with *Helicobacter pylori* and a significantly higher frequency of diffuse intestinal type and poorly differentiated carcinoma, seem to be typical of younger age [10, 11]. However, as data on the biological and clinical courses of gastric cancer in young patients are still controversial, we carried out a retrospective cohort study to point out the reasons for the different biological behavior and prognosis in younger patients. The aim of this study was to identify those independent risk factors influencing mortality, morbidity, and prognosis in patient younger than 50 when compared to older patients and to identify cancer characteristics typical of a younger age.

## 2. Patients and Methods

**Figure 1:**
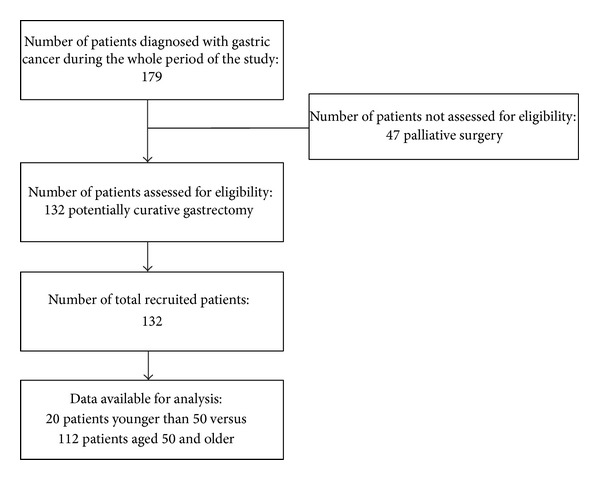
Flowchart of patients with gastric cancer included in the retrospective cohort study.

Over the period from January 1996 to December 2012, a series of 179 consecutive patients were admitted to our surgical department for the treatment of gastric cancer. Among them, 132 patients underwent potentially curative gastrectomy (R0), and they represented the cohort of patients for the current study (Figure 1). The operation was considered to be curative when no grossly visible tumor tissue and no metastatic spread to the liver and peritoneum or lymph node involvement were left and resection margins were free from disease on histological examination.

Patient eligibility for subtotal versus total gastrectomy was usually related to the tumor site and a distance of 6 cm or more from the proximal edge of the tumor to the cardia. Roux-en-Y reconstruction with 40 to 60 cm length of jejunum was used both in case of total and subtotal gastrectomy as reported elsewhere [12]. Modified D2 lymphadenectomy was performed as a standard procedure in most cases. D2 lymph node dissection includes lymph node stations 1–11 at the N2 level in the Japanese classification [13]. Our dissection also included those lymph nodes from the hepatoduodenal ligament, that is, the station 12 part of the N3 level. We identified this dissection as D2 resection, as reported by other authors [14]. A number of patients underwent dissection of the lymph nodes located behind the pancreas (station 13), where the duodenum was mobilized from the inferior vena cava and the aorta. Histological cancer type was classified as intestinal or diffuse following Lauren's histological criteria, and pathologic cancer staging was in accordance with the 6th edition of the pTNM (pathologic tumor, node, metastasis) classification. Five pathologists from the same institution were involved in the evaluation of the surgical specimens throughout the period of study.

### 2.1. Study Design

We carried out a retrospective cohort study to compare 20 patients younger than 50 and 112 patients aged 50 and older in whom gastrectomy was regarded as curative. The study was conducted in accordance with the recommendations in the Strengthening the Reporting of Observational Studies in Epidemiology (STROBE) guidelines for reporting observational studies [15]. All medical records were reviewed retrospectively. The following tests were routinely included in the preoperative staging of gastric cancer patients: serum tumor markers, upper endoscopy with biopsy, *H. pylori* detection, abdominal ultrasonography, and whole-body computed tomography scan. Conversely, endoscopic ultrasonography and positron emission tomography (PET) scan were used only when doubt persisted related to accurate staging. The comparison between the two age groups involved evaluation of patient characteristics, including preoperative abnormalities, tumor characteristics such as site and diameter, histological type, lymph node metastasis, and pathological staging. Data from operative treatment were evaluated with consideration of postoperative morbidity, mortality, and overall and cancer-related survival. Postoperative morbidity was separately analyzed as medical or surgical complications, while postoperative mortality was reported as death by medical or surgical complications.

After discharge, all patients were given a scheduled clinical and instrumental follow-up program in the oncological outpatient department, the mean duration of which was 63.1 months (median 66 and range 2–198 months). Six months after the operation, patients were investigated according to blood count, serum tumor markers, abdominal ultrasonography, and upper endoscopy. Following this, blood count, serum tumor markers, and abdominal ultrasonography were examined every 6 months for 3 years, while whole-body computed tomography and upper endoscopy were performed on a yearly basis for at least 5 years. PET scans were also used where a complement to the anatomical imaging was needed for patient management.

Performance status was assessed in every patient at 6 months after the operation by means of the Karnofsky scale to measure patient autonomy in dealing with normal life postoperatively. The Karnofsky scale is useful for the clinical estimate of physical status, performance, and prognosis after a therapeutic procedure. Patient autonomy is quantified on a scale from 0 (i.e., dead) to 100 (i.e., perfectly well). Data regarding performance status were collected during the course of an interview by a member of the medical team or by phone.

### 2.2. Statistical Analysis and Synthesis of the Results

Data were collected in a planned relational computer database (Microsoft Access), including patient and tumor characteristics. All statistical analyses were carried out using the MedCalc 2011 statistical software (version 11.5.1). Data for age, body mass index (BMI), duration of symptoms, diameter of tumor, postoperative hospital stay, and Karnofsky index were presented as the mean ± standard deviation (SD). Data were compared for statistical analysis using the *χ* ^2^ test to evaluate the differences between qualitative variables and using Student's *t*-test to compare quantitative variables. Overall and actuarial survival (cancer-related) was estimated by the Kaplan-Meier method followed by the log-rank test for comparison of survival rates. For each survival analysis (overall survival and cancer-related survival) both the main event and censoring events were defined.

Another objective of statistical analysis was to identify independent risk factors that were significantly related to the younger age of patients by means of logistic regression analysis, and those independent factors influencing survival were identified using multivariate analysis according to the Cox proportional Hazard models. Differences were considered significant when *P* < 0.05. The *P* values of the study were reported as calculated by statistical software programs, which were always bilateral, that is, *P* = and not *P*< or *P*>.

## 3. Results

### 3.1. Clinicopathological Findings

Younger individuals with gastric cancer represented 15.1% of the whole cohort of patients (20 out of 132). The mean age was significantly different in the two study groups, at 45.8 years (range 27–49) in the younger group versus 68.8 years (range 50–87) in the older group (*P* = 0.000). Gender was not significantly different between groups, even if there was a predominance of female patients in the younger one. Younger patients had significantly less chronic disease and lower American Society of Anesthesiology (ASA) scores than older patients (*P* = 0.049 and *P* = 0.022, resp.). In terms of the symptoms of the disease, younger patients had significantly less preoperative weight loss, longer duration of symptoms, and more frequent association with *H. pylori* infection (*P* = 0.032, *P* = 0.030, and *P* = 0.013, respectively; Table 1).

This study revealed a significant difference regarding the diffuse histological type of gastric cancer, which was more frequently associated with younger patients (*P* = 0.038). No other significant difference was found in terms of pathological characteristics (Table 2).

### 3.2. Surgical Treatment and Chemotherapy

In the younger group, 50.0% of patients (10/20) underwent subtotal gastrectomy as a scheduled procedure because the tumor was located in the lower or middle third of the stomach; 35.0% (7/20) had total gastrectomy by necessity because the tumor was located in the upper or middle third of the stomach. Moreover, regardless of the tumor site, 3 patients (15.0%) in the younger group with a diffuse histological subtype (signet ring cell carcinoma) underwent total gastrectomy as a scheduled procedure because the biological behavior of the tumor was deemed as more aggressive (Table 3).

Among patients aged 50 or older, 58.9% (66/112) underwent subtotal gastrectomy as a scheduled procedure because the tumor was located in the lower or middle third of the stomach; 34.8% (39/112) had total gastrectomy by necessity because the tumor was in the upper third, middle third, or the whole stomach. Finally, regardless of the tumor site, 7 patients (6.3%) in the older group with a diffuse histological subtype underwent total gastrectomy as a scheduled procedure for the same reasons as in the younger group (Table 3).

Overall, gastrectomy with D2 lymphadenectomy was carried out in 118 patients (89.4%), while in 11 patients a D1 lymphadenectomy was performed (the latter subgroup included patients older than 80 years in poor clinical condition). A 60-year-old patient underwent total gastrectomy with splenectomy and distal pancreatectomy by necessity (D4 resection) because the tumor involved the short vessels of the stomach. In both groups, surgery was extended more frequently to the gallbladder, and there was no significant difference between the groups. Currently, we always perform cholecystectomy both in cases of subtotal and total gastrectomy for gastric cancer in order to avoid postoperative cholecystitis or stones due to gallbladder denervation.

Neoadjuvant chemotherapy started with three cycles before surgery and again after surgery. Recommended protocols were as follows: epirubicin, cisplatin, and fluorouracil (ECF), epirubicin, oxaliplatin, and capecitabine (EOX, i.e., Xeloda), and fluorouracil (5-FU)/cisplatin; alternatively, capecitabine was used instead of 5-FU or oxaliplatin instead of cisplatin. Adjuvant chemotherapy started with fluoropyrimidine alone, or alternatively with oxaliplatin (Xelox) in combination with capecitabine.

### 3.3. Early Postoperative Results and Risk Factors Related to Younger Age

The mean postoperative hospital stay was 19.5 days (range: 10–32 days) in the younger group and 18.4 days (range: 10–38 days) in the older one, with a median value of 17 in both groups. The stratification of postoperative hospital stay regarding total and subtotal gastrectomy showed no statistically significant difference between groups (Table 3).

Throughout the period of the study, from January 1996 to December 2012, we found an overall morbidity of 31.0% and a mortality of 6.8% (complications and deaths occurring within 30 days of the operation). The morbidity and mortality rates observed were 35.0% and 5.0% in the younger group and 30.3% and 7.4% in the older one, respectively, and no statistically significant difference was exhibited. There was no significant difference between the incidence of surgical and medical complications in the groups. However, death resulting from medical complications was more frequent in the older group and no patient of the younger group died for medical reasons within 30 days of the operation (Table 4).

Younger patients showed a statistically significant higher risk of having a diffuse histological type of gastric carcinoma and *H. pylori* infection after multivariate analysis by logistic regression (Table 5).

### 3.4. Late Results: Survival, Prognostic Factors, and Performance Status

**Figure 2:**
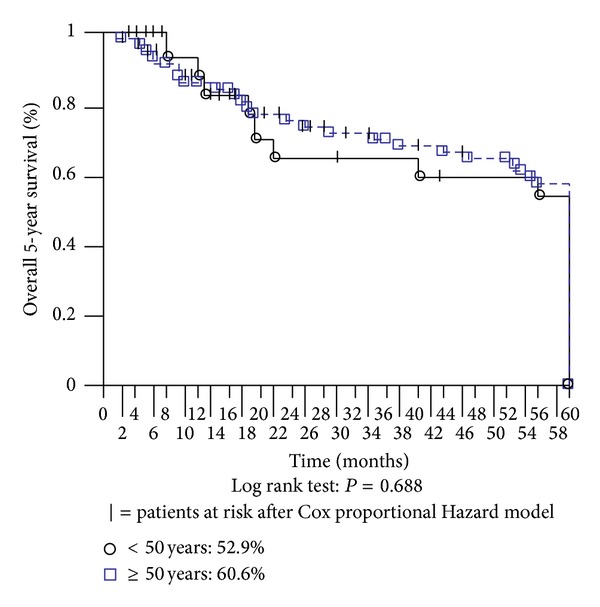
Overall 5-year survival.

The median survival time was 60 months in the younger group (range: 5–143 months) versus 66 months in the older group (range: 2–198 months). In terms of death by surgical or medical complications within 30 days of the operation, one patient in the younger group died due to duodenal dehiscence; moreover, three patients in the older group died because of duodenal dehiscence, wound infection, or gangrenous cholecystitis. One patient in the older group died because of pulmonary embolism, one died of hepatic failure, two died of pulmonary edema, and another one died of stroke (Table 4). After censoring these events, the overall 5-year survival was 52.9% in the younger group and 60.6% in the older one (Figure 2).

**Figure 3:**
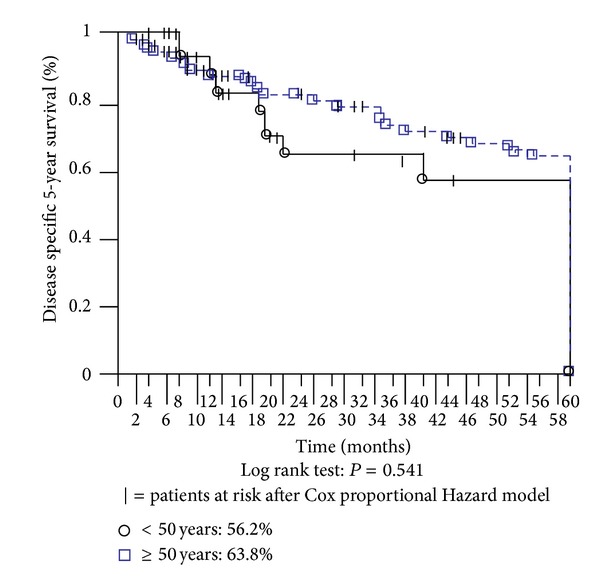
Disease specific 5-year survival.

Over the 5-year follow-up, one patient in the younger group died of stroke; three patients in the older group died of cardiac infarct, two of stroke, and two others of unrelated carcinoma. Excluding these censored events during follow-up, together with death by surgical or medical complications within 30 days of the operation, the two considered age groups showed a similar cancer-related survival rate (Figure 3). In our sample of patients who underwent gastric resection with curative intent, the actuarial 5-year survival was 56.2% in the younger group versus 63.8% in the older one, without a statistically significant difference. Moreover, there was no statistically significant difference between groups in terms of stage-stratified survival (data not shown in figures). On multivariate analysis, advanced cancer stage and diffuse histological type were the prognostic factors influencing cancer-related survival in both groups, regardless of age (Table 6).

In terms of performance status following surgery, although the older patients required more help for personal needs than the younger ones, no significant difference was found between the two groups in terms of activities of daily living assessed by the Karnofsky scale (Table 7).

## 4. Discussion

Gastric cancer is commonly associated with patients over the age of 60, but a significant percentage of those who develop the disease are below 50 years of age [2, 16]. In our series, younger patients with gastric cancer represented 15.1% of the whole cohort of patients, which is a notable proportion of clinical interest. However, several biological and clinical aspects of gastric cancer in young patients still remain a matter of debate.

The main results of our investigation were that younger patients with gastric cancer had significantly fewer comorbidities and more favorable ASA scores than older patients; they had significantly less preoperative weight loss and a significantly longer duration of symptoms before operation; *H. pylori* infection and the diffuse histological type of gastric cancer were significantly associated with younger patients after multivariate analysis. In terms of overall, cancer-related, and stage-related 5-year survival, there was no statistically significant difference between the two age groups; advanced cancer stage and the presence of the diffuse histological type of gastric cancer were the two independent negative prognostic factors influencing cancer-related survival, regardless of age. The survival probability of younger patients did not differ from that of elderly patients provided that curative surgery was performed. Indeed, the similar survival rate in patients aged less than 50 and those aged 50 and older suggests that survival correlates with the above-mentioned prognostic risk factors rather than age.

Our data confirm other studies, in which postoperative mortality mainly correlated to comorbidities of older patients [17, 18]. Although there was no statistically significant difference between groups in terms of performance status as assessed by the Karnofsky scale, the better physical status of younger patients may explain the lower percentage of medical complications and the absence of medical death within 30 days after the operation [5].

The significantly longer duration of symptoms in younger persons is responsible for the delay in diagnosis and hospitalization and for the more advanced tumor stage [16]. These patients are rarely ascribed to a risk group for malignant disease because of their younger age [17]. In those countries where the incidence of gastric cancer is decreasing, it seems that there is no actual reason to enroll younger patients in an endoscopic surveillance program [5]. However, the presence of persisting dyspepsia or alarm symptoms should alert patients and physicians to the need for an urgent endoscopic diagnosis in younger patients [5, 17]. This is not an irrelevant consideration, as the symptoms are often overlooked in young patients and there is a widespread and indiscriminate use of proton pump inhibitors that can change the clinicopathological features of gastric cancer [19].

*Helicobacter pylori* infection is strongly related to the development of gastric cancer in young patients [10, 11, 20, 21]. In the current study, younger patients showed a statistically significant higher risk of having *H. pylori* infection and the diffuse histological type of gastric carcinoma after multivariate analysis. Actually, the diffuse histological type of gastric carcinoma is reported to be typical of younger patients [2, 11, 16].

From the results of the current research and according to other working groups [8, 9, 11], overall, cancer-related, and stage-related 5-year survival were similar in both age groups, and no statistically significant difference was observed. However, there is not a straightforward trend in the literature when it comes to evaluating the survival of younger patients with gastric cancer. Other authors have obtained different results, so that gastric cancer in younger patients has been commonly considered more aggressive and as having a poorer prognosis [5–7, 22]. Conversely, other researchers have reported better long-term survival outcomes in younger age groups [7, 16, 17, 23]. A recent investigation conducted on 2,757 patients younger than 45 showed that younger age was associated with improved survival after stratification by cancer stage at presentation, suggesting that a stage-dependent rather than age-dependent approach should be taken in younger patients with gastric cancer [5]. Age is an accepted prognostic factor after surgery for gastric cancer, and several authors have demonstrated that younger age is an independent negative prognostic factor [7, 22]. Conversely, our experience has shown that advanced cancer stage and the presence of the diffuse histological type of gastric cancer were the only independent negative prognostic factors influencing cancer-related survival, regardless of patient age. However, the 7.6% difference in 5-year cancer-related survival could be a clinically relevant difference between age groups, as extremely expensive oncological treatments providing smaller differences in survival are being applied in patients with worse prognoses than those of our cohort. The sample size in our study and consequently the statistical power of the research may be not adequate to show a lack of statistical difference in survival between groups. Moreover, the results should be verified by long-term follow-up, as the number of patients at risk may change as the follow-up proceeds.

The family history of our gastric cancer patients was also similar in the two age groups. However, almost 10% of all gastric cancer patients may show a family history of neoplasia, while in younger patients, a positive family history may be present in up to 19% of cases [24]. With regard to these considerations, it is believed that gastric carcinogenesis is accelerated in younger patients, suggesting the existence of separate family genetic entities [25]. Among different gastric carcinoma predisposing syndromes, hereditary gastric cancer (HDGD) is caused by a mutation of the E-cadherin gene (CDH1), which carries a more than 70% lifetime gastric cancer risk [26]. Better understanding of gastric carcinogenesis and evidence at a molecular genetics level may be the way forward when it comes to categorizing gastric cancer in the young as a distinctive clinical entity [25].

## 5. Conclusions

*Helicobacter pylori* infection and diffuse histological type of gastric cancer were found to be typical of younger patients in our study. Following the results of the present investigation, however, we do not have sufficient evidence to say that younger patients had a similar survival or to consider gastric cancer in younger patients as a different clinical entity when compared to older patients. Further studies are needed to better understand the progression of carcinogenesis in younger patients and to improve gastric cancer classification.

## Figures and Tables

**Table 1:** Characteristics and preoperative abnormalities of patients with gastric cancer.

Parameter	Patients <50 years (20)	Patients ≥50 years (112)	*P*
Age (years), mean ± SD	45.8 ± 3.6	68.8 ± 10.2	0.000
Range	27–49	50–87	
Median	48	70	
Sex: female (%)	12 (60.0%)	49 (43.7%)	0.272
Anemia: hemoglobin < 10 g/dL	3 (15.0%)	23 (20.5%)	0.789
Hypoalbuminemia < 3.5 g/dL	5 (25.0%)	50 (44.6%)	0.163
Comorbidities			
Cardiac disease	0 (0%)	17 (15.2%)	0.132
Hypertension	3 (15.0%)	38 (34.0%)	0.155
Previous stroke	0 (0%)	8 (7.1%)	0.469
Chronic obstructive pulmonary disease	1 (5.6%)	15 (13.4%)	0.492
Diabetes mellitus	0 (0%)	7 (6.2%)	0.544
Cirrhosis	0 (0%)	4 (3.6%)	0.881
Number of concomitant disease			
0	15 (75.0%)	54 (48.2%)	0.049
1	4 (20.0%)	34 (30.4%)	
2	1 (5.0%)	17 (15.2%)	
3	0 (0%)	7 (6.2%)	
ASA score			
I-II	19 (95.0%)	75 (67.0%)	0.022
III-IV	1 (5.0%)	37 (33.0%)	
BMI (body mass index)	22.8 ± 4.5	24.2 ± 4.7	0.242
Median	22	24	
Symptoms			
Epigastric pain	12 (60.0%)	75 (67.0%)	0.727
Vomiting	3 (15.0%)	9 (8.0%)	0.565
Dysphagia	2 (10.0%)	14 (12.5%)	0.955
Fatigue	6 (30.0%)	41 (36.6%)	0.552
Loss of appetite	6 (30.0%)	28 (25.0%)	0.847
Sarcophobia	3 (15.0%)	10 (8.9%)	0.666
Upper gastrointestinal bleeding	1 (5.0%)	8 (7.2%)	0.896
Weight loss	6 (30.0%)	66 (58.9%)	0.032
Duration of symptoms (months) mean ± SD	14.5 ± 4.8	7.8 ± 0.7	
Median	10	5	0.030
History of peptic ulcer	1 (5.0%)	3 (2.7%)	0.876
Family history of gastric cancer	1 (5.0%)	7 (6.2%)	0.770
*Helicobacter pylori* infection	5 (25.0%)	6 (5.4%)	0.013

SD: standard deviation; ASA: American Society of Anesthesiology; BMI: body mass index; *P* values are bilateral.

**Table 2:** Pathologic characteristics of operated gastric tumors.

Parameter	<50 years (20)	≥50 years (112)	*P*
Mean tumor diameter (cm) ± SD	4.7 ± 2.6	4.3 ± 2.3	0.461
Median	4	4	
Tumor site			
Upper third	4 (20.0%)	23 (20.5%)	0.806
Middle third	4 (20.0%)	27 (24.1%)	
Lower third	12 (60.0%)	60 (53.6%)	
Whole stomach	0 (0%)	2 (1.8%)	
Depth of cancer invasion			
Early	4 (20.0%)	15 (13.4%)	0.667
Advanced	16 (80.0%)	97 (86.6%)	
Histological type (Lauren's criteria)			
Intestinal	6 (30.0%)	65 (58.0%)	0.038
Diffuse	14 (70.0%)	47 (42.0%)	
Lymph node metastasis			
Positive	13 (65.0%)	63 (56.2%)	0.629
Negative	7 (35.0%)	49 (43.8%)	
Stage TNM 6th edition			
I	7 (35.0%)	38 (34.9%)	0.871
II	2 (10.0%)	31 (27.7%)	
III	6 (30.0%)	31 (27.7%)	
IV	5 (25.0%)	12 (10.7%)	

SD: standard deviation; TNM: tumor node metastasis; *P* values are bilateral.

**Table 3:** Surgical treatment, length of postoperative hospital stay, and chemotherapy.

Parameter	<50 years (20)	≥50 years (112)	*P*
Gastric resection			
Total gastrectomy by necessity	7 (35.0%)	39 (34.8%)	0.513
Total gastrectomy as scheduled procedure	3 (15.0%)	7 (6.3%)	0.359
Subtotal gastrectomy as scheduled procedure	10 (50.0%)	66 (58.9%)	0.618
Lymphadenectomy			
<D2	3 (15.0%)	11 (9.8%)	0.765
≥D2	17 (85.0%)	101 (90.2%)	
Combined resection			
Distal esophagectomy	—	6 (5.3%)	0.682
Distal pancreatectomy	—	1 (0.9%)	
Cholecystectomy	7 (35.0%)	35 (31.2%)	
Appendectomy	—	1 (0.9%)	
Splenectomy	—	1 (0.9%)	
Liver resection for angioma	—	2 (1.8%)	
Left adrenalectomy for adenoma	—	1 (0.9%)	
Postoperative hospital stay (days) ± SD			
Overall	19.5 ± 6.8	18.7 ± 8.2	0.606
Range	10–32	10–38	
Median	17	17	0.435
Total gastrectomy	20.1 ± 4.2	19.2 ± 6.1	
Median	19	18	0.734
Subtotal gastrectomy	17.4 ± 5.0	18.0 ± 8.2	
Median	17	16	
Chemotherapy			
Yes	5 (25.0%)	24 (21.4%)	
No	15 (75.0%)	88 (78.6%)	0.950
Neoadjuvant	1 (5.0%)	—	0.329
Adjuvant	4 (20.0%)	24 (21.4%)	0.878

SD: standard deviation; *P* values are bilateral.

**Table 4:** Postoperative morbidity and related death.

Complications	<50 years (20)		≥50 years (112)		*P*
	Morbidity	Mortality	Morbidity	Mortality	
Surgical					
Anastomotic dehiscence	0	0	1 (0.9%)	0	
Anastomotic stenosis	1 (5.0%)	0	2 (1.8%)	0	
Duodenal dehiscence	0	1 (5.0%)	1 (0.9%)	1 (0.9%)	
Wound infection	1 (5.0%)	0	4 (3.6%)	1 (0.9%)	
Bleeding	1 (5.0%)	0	2 (1.8%)	0	
Gangrenous cholecystitis	0	0	0	1 (0.9%)	
Pancreatitis	1 (5.0%)	0	0	0	
Medical					
Pulmonary embolism	0	0	0	1 (0.9%)	
Pulmonary edema	0	0	3 (2.7%)	2 (1.8%)	
Atelectasis	1 (5.0%)	0	6 (5.4%)	0	
Pleuric effusion	2 (10.0%)	0	10 (9.0%)	0	
Hepatic failure	0	0	0	1 (0.9%)	
Ascites	0	0	4 (3.6%)	0	
Stroke	0	0	0	1 (0.9%)	
Decubitus ulcer	0	0	1 (0.9%)	0	
Overall	7 (35.0%)	1 (5.0%)	34 (30.3%)	8 (7.4%)	
Death by surgical complications	—	1 (5.0%)	—	3 (2.7%)	0.881
Death by medical complications	—	0	—	5 (4.5%)	0.743
Overall death	—	1 (5.0%)	—	8 (7.4%)	0.896

*P* values are bilateral.

**Table 5:** Independent risk factors for gastric cancer related to patients younger than 50 after multivariate logistic regression analysis.

Risk factor	*P*	OR	95% CI
Diffuse histologic type	0.023	11.937	1.386–102.744
*Helicobacter pylori *	0.037	6.358	1.110–36.420

OR: odds ratio; CI: confidence interval; *P* values are bilateral.

**Table 6:** Independent risk factor influencing survival after multivariate analysis by Cox proportional hazard models.

Risk factor	*P*	HR	95% CI
Diffuse histologic type	0.011	14.282	1.840–110.855
Advanced cancer stage	0.047	3.541	1.020–12.289

HR: hazard ratio; CI: confidence interval; *P* values are bilateral.

**Table 7:** Performance status assessed by Karnofski scale (6 months after the operation).

Parameter	Patients <50 years (20)	Patients ≥50 years (112)	*P*
Karnofski index, mean ± SD	80 ± 13.6	69.8 ± 21.5	0.085
Range	60–100	20–100	
Median	80	70	

SD: standard deviation; *P* value is bilateral.
